# The protective role of muscone in the development of COPD

**DOI:** 10.3389/fimmu.2025.1508879

**Published:** 2025-02-17

**Authors:** Tiantian Feng, Xiaolong Guo, Wei Chen, Yanying Zhang, Runjing Dai, Yinfang Zhang, Yongqi Liu, Yiya Liu, Peng Song, Jingchun Fan

**Affiliations:** ^1^ School of Public Health, Centre for Evidence-Based Medicine, Gansu University of Chinese Medicine, Lanzhou, Gansu, China; ^2^ Quality Assurance Department, Lanzhou Institute of Biological Products Co., Ltd, Lanzhou, Gansu, China; ^3^ First Clinical Medical College, Gansu University of Chinese Medicine, Lanzhou, Gansu, China; ^4^ Hospital Infection‐Control Department, Xi’an Aerospace General Hospital, Xi’an, Shanxi, China; ^5^ Experiment and Achievement Transformation Center, Affiliated Hospital of Gansu University of Chinese Medicine, Lanzhou, Gansu, China; ^6^ Key Laboratory of Dunhuang Medicine, Ministry of Education, Gansu University of Chinese Medicine, Lanzhou, Gansu, China; ^7^ School of Public Health, Gansu Medical College, Pingliang, Gansu, China

**Keywords:** muscone, COPD, interleukin-38, cigarette smoke, lipopolysaccharide

## Abstract

**Background:**

Muscone, a key component of musk, exhibits anti-inflammatory properties. However, its therapeutic potential in inflammatory lung diseases, such as chronic obstructive pulmonary disease (COPD), remains largely unexplored. This study aimed to investigate whether Muscone could exert a protective effect in a mouse model of COPD *in vivo*.

**Methods:**

A COPD animal model was established by exposing mice to cigarette smoke (CS) and administering lipopolysaccharide (LPS) intranasally. After 4 weeks, mice were treated daily with dexamethasone (DEX) or different doses of Muscone for 3 weeks. Mouse body weight, lung function, and histopathology were determined. Serum levels of cytokines (IL-38, IL-1β, IL-17, TGF-β, IFN-γ) were measured using ELISA and qRT-PCR. Lung expression of CXCR3, IFN-γ, IL-17A, and RORγt was assessed by immunofluorescence.

**Results:**

The body weight of COPD mice was significantly lower than that of Muscone-treated COPD mice, consistent with decreased lung function, accompanied by reduced circulating and lung IL-38 levels. After Muscone administration, lung function was significantly improved, accompanied by upregulation of circulating and lung anti-inflammatory cytokines, including IL-38, in a dose-dependent manner, while the expression of pro-inflammatory cytokines was significantly reduced. Additionally, Muscone significantly inhibited the protein expression of CXCR3, IFN-γ, IL-17A, and RORγt in lung tissues of COPD mice.

**Conclusion:**

This study demonstrates that Muscone improves lung function in mice with COPD, potentially through a mechanism that may involve the modulation of cytokine expression, including the potential upregulation of anti-inflammatory cytokines such as IL-38. The precise underlying mechanisms of Muscone’s therapeutic effects in COPD remain to be fully elucidated. Further research is needed to investigate the correlation between COPD lung pathophysiology and the specific effects of Muscone treatment, including a more detailed analysis of the balance between pro- and anti-inflammatory mediators in COPD animal models, particularly utilizing IL-38 GKO mice to further investigate the role of IL-38 in mediating the therapeutic effects of Muscone.

## Introduction

Chronic obstructive pulmonary disease (COPD) is a major global health concern characterized by persistent airflow limitation and chronic inflammation (1, 2). The incidence and mortality rates of COPD are rising annually worldwide, emphasizing its significant public health impact (3). The pathogenesis of COPD involves complex mechanisms, including airway and lung inflammation, an imbalance between proteases and anti-proteases, an imbalance in oxidation and antioxidant processes, and reduced immune function (4, 5).

Regular smoking and exposure to environmental pollutants, such as dust particles and toxic gases, can induce inflammation within the body, contributing to the development of COPD (6, 7). Consequently, inhibiting the inflammatory response is a crucial therapeutic strategy for this condition.

Current COPD treatment often involves a combination of bronchodilators, corticosteroids, antibiotics, expectorants, antioxidants, and immunomodulators (8–14). However, multidrug therapy can lead to serious side effects due to complex pharmacokinetics (15). Therefore, there is an urgent need to develop novel, safe, and effective medications for COPD management.

Muscone, the primary component of musk, exhibits anti-inflammatory, anti-cancer, and anti-tumor properties (16–18). It modulates various cellular processes, including inflammation, apoptosis, and angiogenesis (19). Muscone has been shown to inhibit macrophage activation and improve ventricular remodeling after myocardial infarction (20). Furthermore, studies have demonstrated that muscone can suppress the activation of the NLRP3 inflammasome and NF-κB, resulting in reduced mRNA levels of inflammatory factors such as IL-1β, IL-6, and TNF (21, 22).

Inflammation plays a pivotal role in COPD pathogenesis (23). Exposure to harmful particles triggers immune responses in the respiratory tract, leading to the activation of immune cells and the release of pro-inflammatory cytokines, such as TNF, IL-6, IL-8, and MMPs. These mediators disrupt alveolar structure, leading to persistent inflammation and tissue damage, which ultimately contributes to COPD development (24–26). While IL-38, a member of the IL-1 family, exhibits anti-inflammatory properties, its specific role in COPD remains to be fully elucidated (27, 28).

The therapeutic potential of Muscone in inflammatory lung diseases, such as COPD, remains largely unexplored. This study aimed to investigate whether Muscone could exert a protective effect in a mouse model of COPD.

## Methods

### Mice

C57BL/6J male mice, aged 6 weeks (n=60), were procured from Spearfish Biotechnology Co. (Beijing). All animals were housed in a specific pathogen-free facility maintained at 22°C with 40-50% humidity, on a 12-hour light/dark cycle, and had ad libitum access to standard laboratory food. Ethical approval for this study was granted by the *Animal Experimentation Ethics Committee of Gansu University of Traditional Chinese Medicine* (approval number: SY2023-956). The mice were randomly assigned to either the COPD or normal group. The number of mice used was determined based on prior experimental knowledge and relevant publications. CS exposure was administered by research technicians who were blinded to the study conditions.

### Establishment of COPD animal model

**Figure 1:**
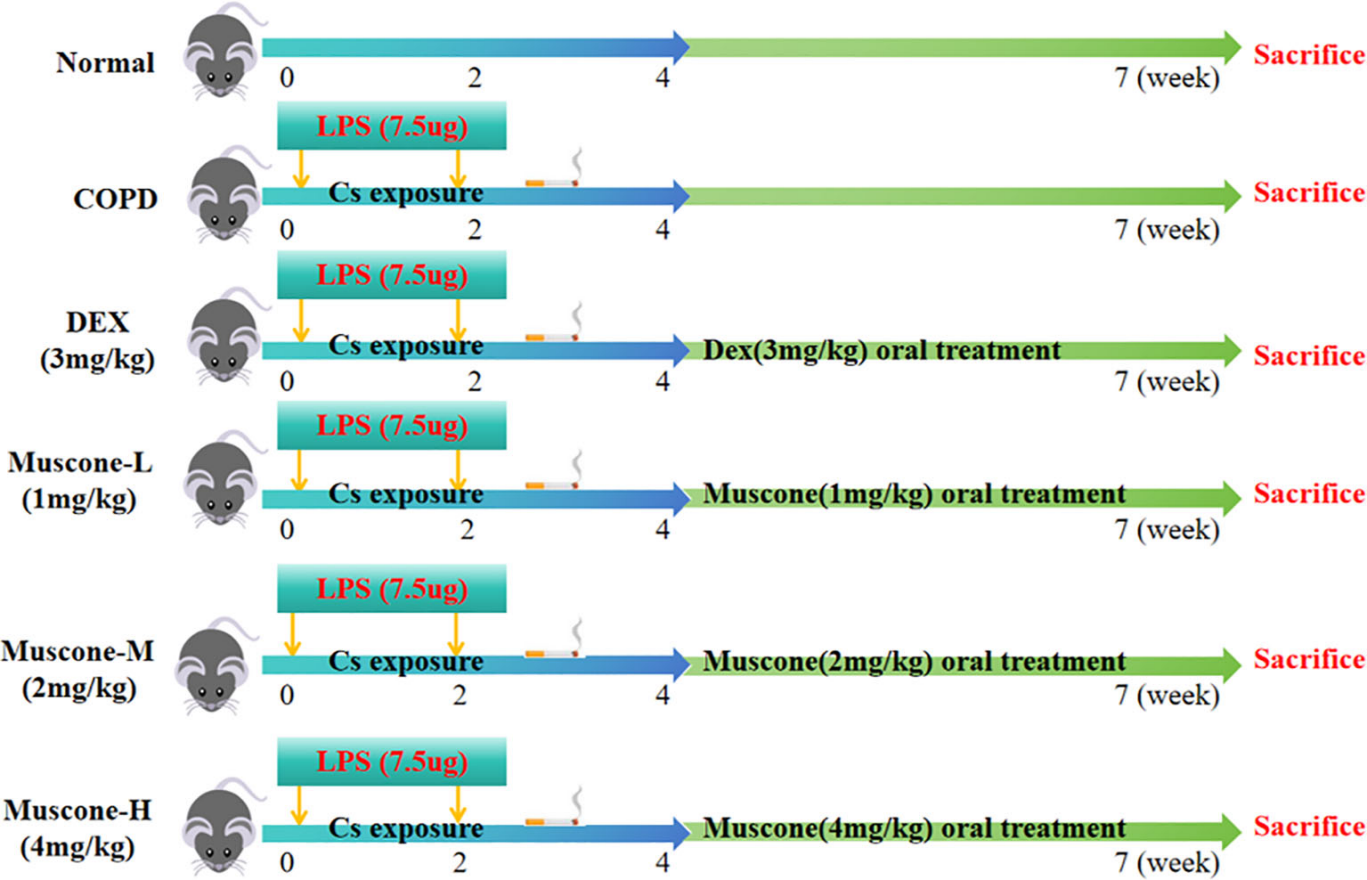
Timeline of the experimental protocol.

The COPD mouse model was established using the CS+LPS induction method. Age-and sex-matched 8-week-old male mice (n=10 per group) were randomly divided into six groups. The normal group was housed in a smoke-free environment, while the remaining mice underwent intra-tracheal instillation of LPS (7.5 μg in 50 μl of saline; L8880; Solar bio, China) on days 1 and 14. With the exception of days 1 and 14, the mice were placed in a smoke box (dimensions: 50 cm x 60 cm x 90 cm) for passive inhalation of cigarette smoke (Lanzhou brand cigarettes, tar: 13 mg; nicotine: 1.3 mg). The exposure regimen consisted of 10 cigarettes per hour, with each session lasting 2 hours (smoke concentration: 800-1000 ppm), and a ventilation interval of 15 minutes each hour. This procedure occurred twice daily, 6 days a week, concluding on week 4 (**Figure 1**). On week 5, one mouse from each group was sacrificed for pathological diagnosis to confirm the successful establishment of the model. To establish the COPD animal model, we used a combination of cigarette smoke exposure and LPS intra-tracheal instillation. Once COPD was established, both smoke exposure and LPS intra-tracheal instillation were stopped. To assess the effects of Muscone, the COPD animals were given Muscone via gavage, while the negative control group received normal saline and the positive control group was given DEX via gavage.

### Dosing method

According to the literature method, mice in the treatment group received muscone intragastrically at dosages of 1, 2 and 4 mg/(kg/d) (29), while the positive control group was administered 3 mg/(kg/d) of DEX (30). Mice in the normal and COPD model groups were given equal amounts of saline by gavage. Each treatment group was administered once daily for 3 weeks starting at week 5. Notably, there were no fatalities during the administration period. The modeling and drug delivery process is illustrated in **Figure 1**.

### Pulmonary function measurement

Lung function was assessed using a small animal spirometer at the end of week 7 (Best lab; Anirec2005; China), to which the mouse was connected and mechanically ventilated. The lung function test commenced once the respiratory rhythm of the mouse synchronized with that of the ventilator. An average breathing frequency of 150 breaths per minute was established. Various pulmonary function parameters were measured, including Inspiratory Time (Ti), Expiratory Time (Te), Peak Inspiratory Flow (PIF), Peak Expiratory Flow (PEF), Tidal Volume (TV), and Minute Ventilation Volume (MV). Following the lung function assessments, the mice were euthanized through blood collection via the abdominal aorta.

### Lung preparation

The left lung tissues were then immediately fixed in 4% formaldehyde (Bio sharp, Hefei, China) for 48 hours. Paraffin sections (4μm) were prepared and stained with hematoxylin and eosin (H&E), and were also subjected to immunofluorescence staining, as described (31, 32). The right lung tissues from each group of mice were also removed and stored at -80°C for qRT-PCR.

### Pulmonary histopathological analysis

For histopathological examinations, tissue sections were prepared as previously described (32). Briefly, lung tissues were fixed using 10% formalin, then embedded within paraffin, and then cut into 4-μm sections. Tissue sections were stained with H&E for histological analysis.

### RNA extraction

Total RNA was extracted from the lungs using the following procedure: the trachea and lungs were resected, and the airways were carefully separated from the lung parenchyma with sterile forceps. The lungs were then snap-frozen and stored at -80°C. Subsequently, the tissue was thawed in sterile PBS (Solarbio, Beijing, China). Total RNA was extracted using Trizol reagent (Ambion, Texas, USA) in accordance with the manufacturer’s instructions and stored at -80°C.

### Quantitative real-time PCR

RNA was reverse transcribed into cDNA strands using the PrimeScript™ RT Reagent Kit with gDNA Eraser (Yeasen, Shanghai, China). Quantitative reverse transcription PCR was conducted on the CFX96–C1000 system (Yeasen, Shanghai, China) utilizing the SsoFast™ EvaGreen^®^ Supermix kit (Yeasen, Shanghai, China). Three replicate tests were performed for each sample, and glyceraldehyde-3-phosphate dehydrogenase (GAPDH) was detected as an internal reference. Employing the 2−ΔΔCt method for quantitative analysis.

The PCR primers used in this study were as follows:

IL-38:

5’-CCAAAGGCTCCATGTGGTTG-3’ (forward)

5’-AGGAGGGCAAGGTTAATGG-3’ (reverse);

TNF:

5’ -CCCTCACACTCACAAACCAC-3’ (forward)

5’-ATAGCAAATCGGCTGACGGT-3’ (reverse);

IL-1β:

5’-TGGCAACTGTTCCTGAACTC-3’ (forward)

5’-AGTGATACTGCCTGCCTGAAG-3’ (reverse);

NLRP3:

5’-TGTCAGGATCTCGCATTGG-3’ (forward)

5’-AGTAAGGCCGGAATTCACC-3’ (reverse);

GAPDH:

5’-TGTTTCCTCGTCCCGTAG-3’ (forward)

5’-CAATCTCCACTTTGCCACT-3’ (reverse).

The real-time PCR results were analyzed using the Applied Biosystems 7500 Real-Time PCR System software (Applied Biosystems, CA, USA), and the fold change in cDNA expression of the target gene relative to the endogenous control (GAPDH) was calculated using the 2−ΔΔCt method.

### Enzyme-linked immunosorbent assay

The mouse blood was centrifuged at 4°C, 3500 rpm for 10 min, and the serum was stored at-80 °C for ELISA detection. Commercial ELISA kits (CUS Ag, Wuhan, China) were used to measure the following analyses in duplicate from mouse serum samples, following the manufacturer’s instructions: IL-38 (ZC-10406), IL-1β (ZC-10247), IL-17 (ZC-10243), TGF-β (ZC-10401), IFN-γ (ZC-10280), VEGF (ZC-10379), and TNF (ZC-10225). Equal amounts of total protein were loaded into each well. Absorbance at 450 nm was measured using an Enspire enzyme marker (Perkin Elmer, Waltham, USA).

### Immunofluorescence staining

The sections (4μm) were dew axed, rehydrated, and antigen retrieved as described (31, 32). Sections were permeabilized with 0.5% Triton X-100 for 15 min at room temperature (RT) and rinsed three times with PBST. The sections were blocked with normal goat serum (E-IR-R110) from the kit (Elabscience, Wuhan, China) for 30 min. Then stained with anti-CXCR3, anti-IL-17A (green fluorescence) and anti-IFN-γ, anti-RORγt (red fluorescence), where the dilutions of anti-CXCR3, anti-IL-17A, anti-IFN-γ, and anti-RORγt were 1:500, 1:100, 1:200, and 1:500, respectively (Abcam, London, UK), and incubated at 4°C overnight. The sections were incubated overnight. The sections were rinsed three times with PBST for 3-5 minutes each time. Diluted fluorescent labelled secondary antibodies were then added to the wet kit and incubated in the dark at RT for 60 min. DAPI stain (E-IR-R103) from the kit was added drop wise and the nuclei were stained for 5 min in the dark. Finally, anti-fluorescence quenching mounting solution (E-IR-R119) was applied to the slides, and then cover slides, making sure to avoid light exposure after secondary antibody incubation. The slides were imaged using a light microscope (Leica, Wetzlar, Germany).

### Statistical analysis

All analyses were performed using GraphPad Prism 8 (San Diego, CA, USA). Normally distributed measurements are presented as mean ± standard deviation, with a t-test used for comparisons between two groups. For comparisons involving multiple groups, one-way ANOVA was performed, followed by Dunnett’s test for *post-hoc* multiple comparisons. Non-normally distributed measures are reported as M (P_25_, P_75_), with the Mann-Whitney U test used for comparing two independent samples. The Kruskal-Wallis H test was applied for comparisons among multiple groups. A *P* value < 0.05 was considered significant.

## Results

### General appearance of mice

Normal group animals showed shiny fur, even breathing, and normal activity during the entire experimental period. The COPD group animals displayed dull fur, partial hair loss, irritability, and a tendency to huddle together following CS+LPS treatment. In addition, these animals also showed fatigue and loud breathing. There was no death of animals during the entire experimental period. The sick appearance was much improved following DEX treatment, as well as muscone treatments.

### Mouse weight and pulmonary dysfunction in COPD mice

**Figure 2:**
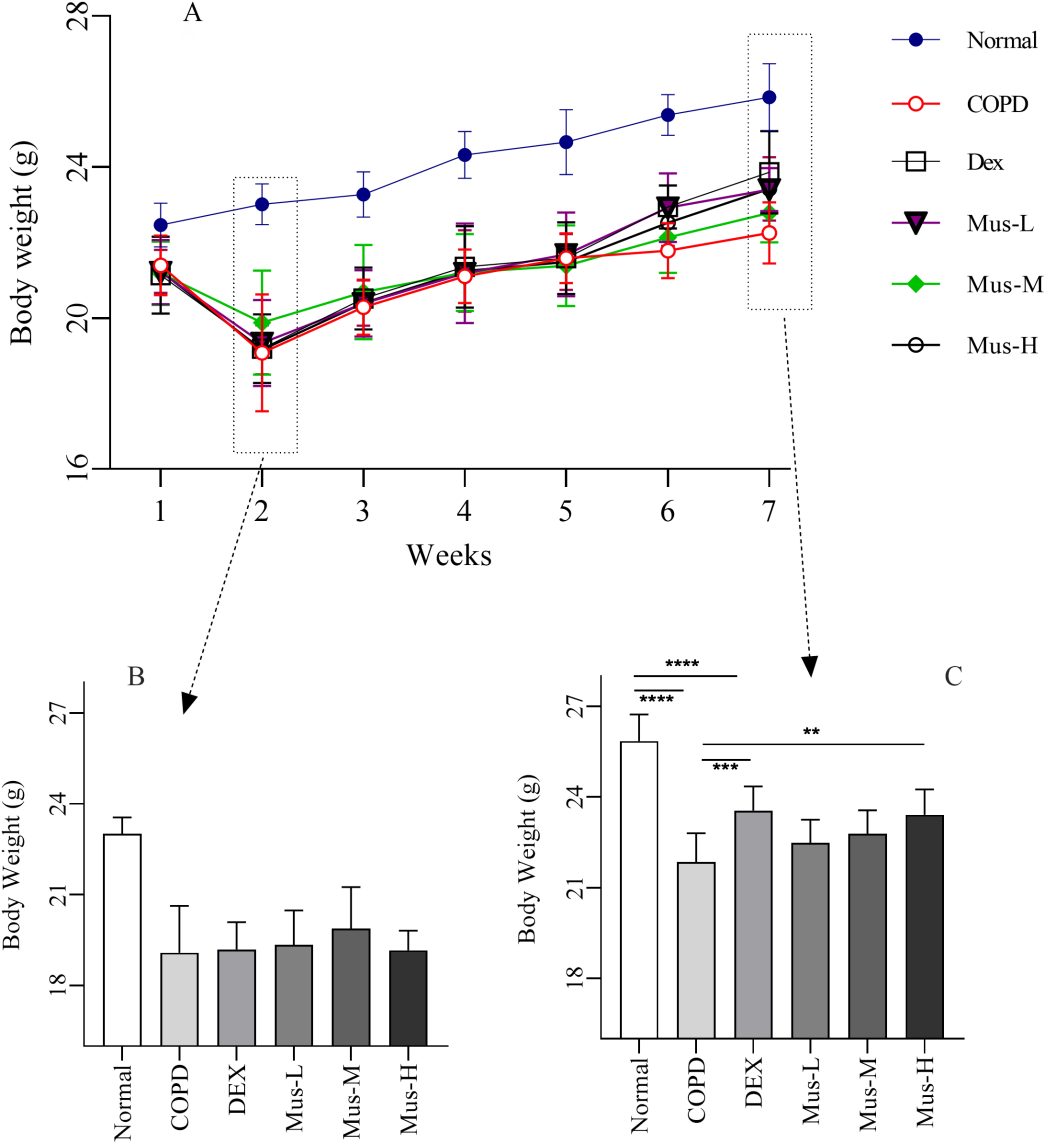
The body weight change of COPD mouse model **(A-C)**. ****P < 0.0001, ***P < 0.001, **P < 0.01.

Mice in each group gradually gained weight from modeling to drug administration (1-7 weeks) (**Figure 2A**). There was significant body weight (~18%) lost over 2 weeks COPD model (CS+LPS treatment) development, compared with the normal animals (**Figure 2B**). As expected, the body weight of of COPD animals wasn’t changing much immediately following DEX, Mus-L, Mus-M, or Mus-H individuals. Furthermore, the body weight of all the experimental animals was gradually increased toward week 6 (**Figure 2C**).

Although there was also gradual body weight gain over the following 5 weeks, the overall body weight was significantly lower (~15%) from the COPD animals, compared to that of the normal groups (*p* < 0.001) (**Figure 2C**). As expected, the body weight of the DEX treated COPD group seemed to be improved, from the second week of the treatment, and much noticeable at the week 3 post treatment (*p* < 0.001) (**Figure 2C**). Subsequently, the improvement of the body weight was only observed from the Mus-H treated COPD animals (*p* < 0.01) (**Figure 2C**), but not from Mus-L or Mus-M treatment.

**Figure 3:**
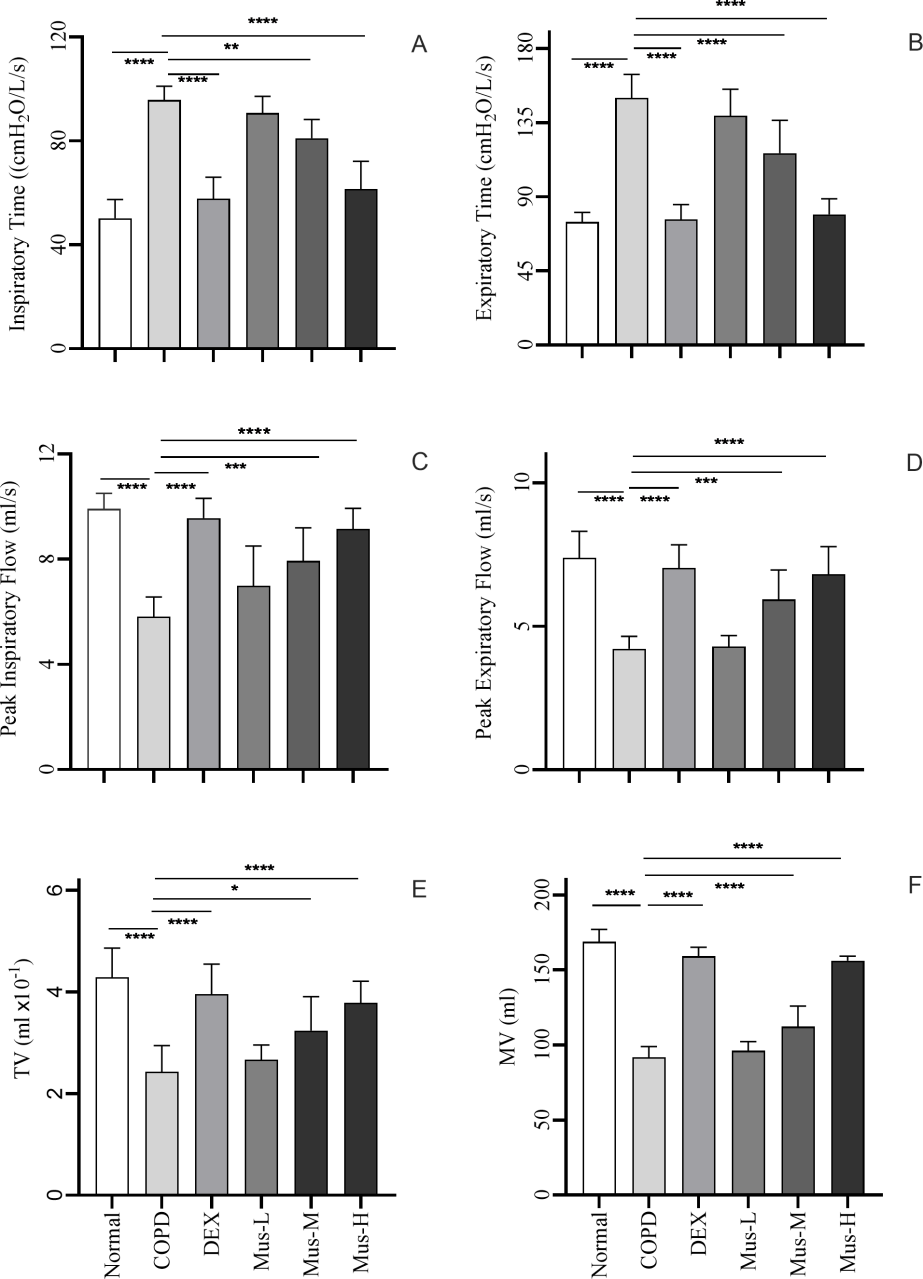
Indicators of lung function i each group of mice **(A-F)**. ****P < 0.0001, ***P < 0.001, **P < 0.01, *P < 0.5.

There were significant increased nearly twofold Ti (*p* < 0.0001) (**Figure 3A**) and Te (*p* < 0.0001) (**Figure 3B**) increased from the COPD group compared to that in the normal group, a result that further validates the reliability of the COPD model. As expected, DEX could resure the increased Ti and Te in the COPD animals. Additionally, muscone could also resure Ti and Te in a dose dependent manner, particularly in the high dosage.

Furthermore, there was a significant reduction in PIF (*p* < 0.001) (**Figure 3C**), PEF (*p* < 0.001) (**Figure 3D**), TV (*p* < 0.001) (**Figure 3E**) and MV (*p* < 0.001) (**Figure 3F**), were significantly reduced by ~50% in COPD group compared to the normal group. Such reduced MV, TV, PIF and PEF were partially restored following DEX treatment or with muscone, also in a dose dependent manner (See **Table 1** for details).

**Table 1:** Comparison of lung function indexes of mice in each group ( $\bar{x}$ ± S, N = 10).

Group	n	Ti (cmH_2_O/L/s)	Te (cmH_2_O/L/s)	PIF (ml/s)	PEF (ml/s)	TV (ml×10^-1^)	MV (ml)
Normal	10	50.20 ± 7.30	74.70 ± 5.74	9.91 ± 0.59	7.38 ± 0.93	4.29 ± 0.57	168.80 ± 8.24
Model	10	95.70 ± 5.36^####^	150.00 ± 14.31^####^	5.80 ± 0.76^####^	4.32 ± 0.32^####^	2.43 ± 0.52^####^	91.70 ± 7.21^####^
DEX	10	57.80 ± 8.28^****^	76.3.10 ± 9.0^****^	9.57 ± 0.75^****^	7.03 ± 0.81^****^	3.96 ± 0.59^****^	159.10 ± 5.97^****^
Mus-L	10	71.90 ± 10.35^####****^	139.10 ± 16.09^####^	6.99 ± 1.51^####^	4.30 ± 0.38^####^	2.67 ± 0.29^###^	96.10 ± 6.26^####^
Mus-M	10	68.20 ± 7.25^##**^	116.50 ± 19.87^####****^	7.94 ± 1.26^###***^	5.94 ± 1.03^##***^	3.24 ± 0.67^###*^	112.30± 13.64^####****^
Mus-H	10	61.40 ± 10.73^****^	79.30 ± 9.45^****^	9.16 ± 0.80^****^	6.82 ± 0.96^****^	3.79 ± 0.42^****^	156.20 ± 2.97^#****^

# indicates that compared to the Normal group, ^####^P < 0.0001, ^###^P < 0.001, ^##^P < 0.01, ^#^P < 0.5.

* indicates that compared to the COPD group, ****P < 0.0001, ***P < 0.001, **P < 0.01, *P < 0.5.

### Muscone inhibits CS+LPS-induced lung injury in mice

**Figure 4:**
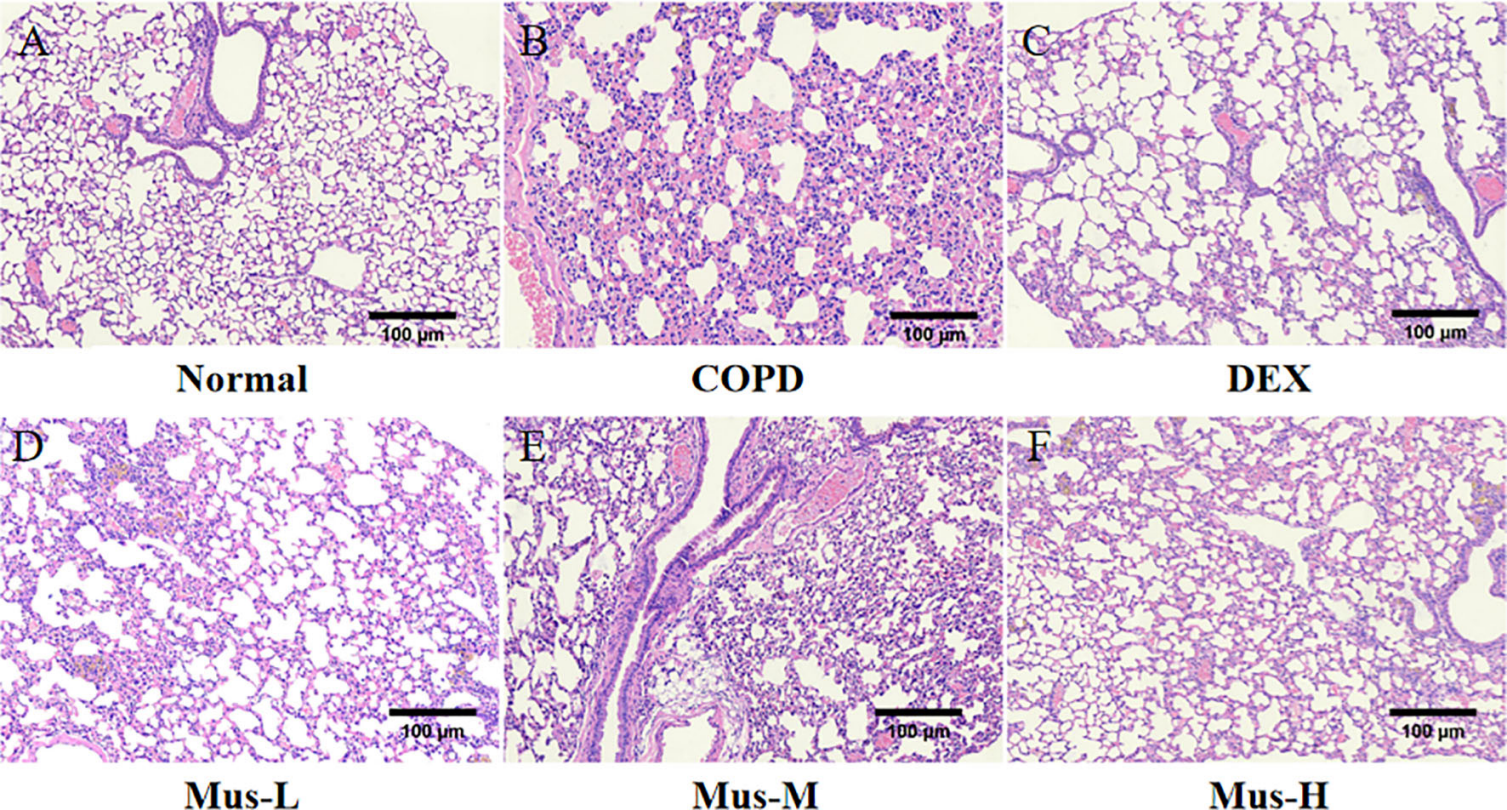
The pathological changes of lung in each group **(A-F)**.

The induction of COPD in the animals was confirmed using histopathology. Compared with the normal group (**Figure 4A**), the lung tissues of mice in the COPD group exhibited severe damage, including large numbers of infiltrating leukocytes, erythrocytes, and effusions in the interstitial tissues (**Figure 4B**). As expected, there was a noticeable histopathological improvement in the lungs of COPD animals following DEX treatment, with a reduction of infiltrating leukocytes and red blood cells by almost half, along with decreased fluid accumulation (**Figure 4C**). Muscone also reduced the severity of COPD in a dose-dependent manner, with higher doses showing better histopathological outcomes (**Figures 4D–F**).

### Muscone promoted mRNA expression of IL-38 in COPD mice

**Figure 5:**
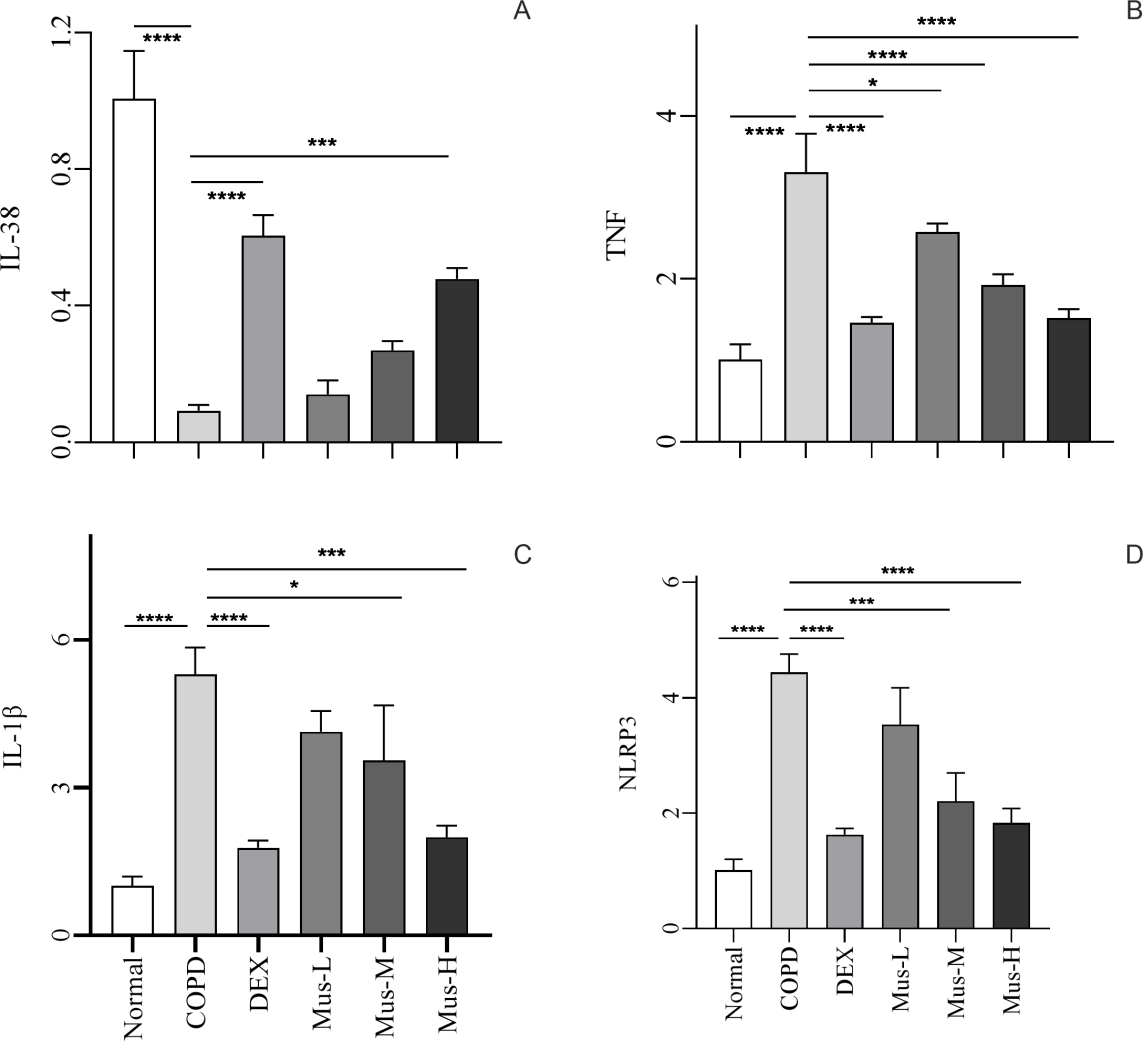
The mRNA expression of IL-38, TNF, IL-lβ and NLRP3 in lung tissue of mice in each group **(A-D)**. ****P < 0.0001, ***P < 0.001, *P < 0.5.

To determine the potential role of IL-38 in the development of COPD in response to the different treatments (**Figure 5A**), IL-38 mRNA was evaluated using qRT-PCR. Constitutive expression of IL-38 was significantly reduced by 90% in the lungs of mice in the COPD group compared with the normal group, which is consistent with the histopathology of the COPD model (*p* < 0.001). DEX treatment reversed this inhibition of IL-38 mRNA expression in the COPD lungs (*p* < 0.001). Similarly, IL-38 mRNA expression was also rescued in response to muscone treatment in a dose-dependent manner.

### Muscone inhibits mRNA expression of TNF, IL-1β, and NLRP3 in COPD mice

To investigate the involvement of pro-inflammatory mediators, the expression of TNF, IL-1β, and NLRP3 mRNA in the lungs from the different treatment groups was assessed (**Figures 5B–D**). TNF mRNA expression was up-regulated nearly threefold in lung tissues of mice in the COPD group, supporting the establishment of COPD (*p* < 0.0001). However, after DEX treatment, the increase in TNF was significantly suppressed, with a reduction of approximately 60% (*p* < 0.0001). Additionally, muscone also reduced TNF expression in the lungs of COPD animals in a dose-dependent manner.

It was not surprising that the expression of the other two inflammatory mediators, IL-1β and NLRP3, was also upregulated in the lungs of COPD animals and could be inhibited by both DEX and muscone in a dose-dependent manner.

### Effect of muscone on serum cytokines

**Figure 6:**
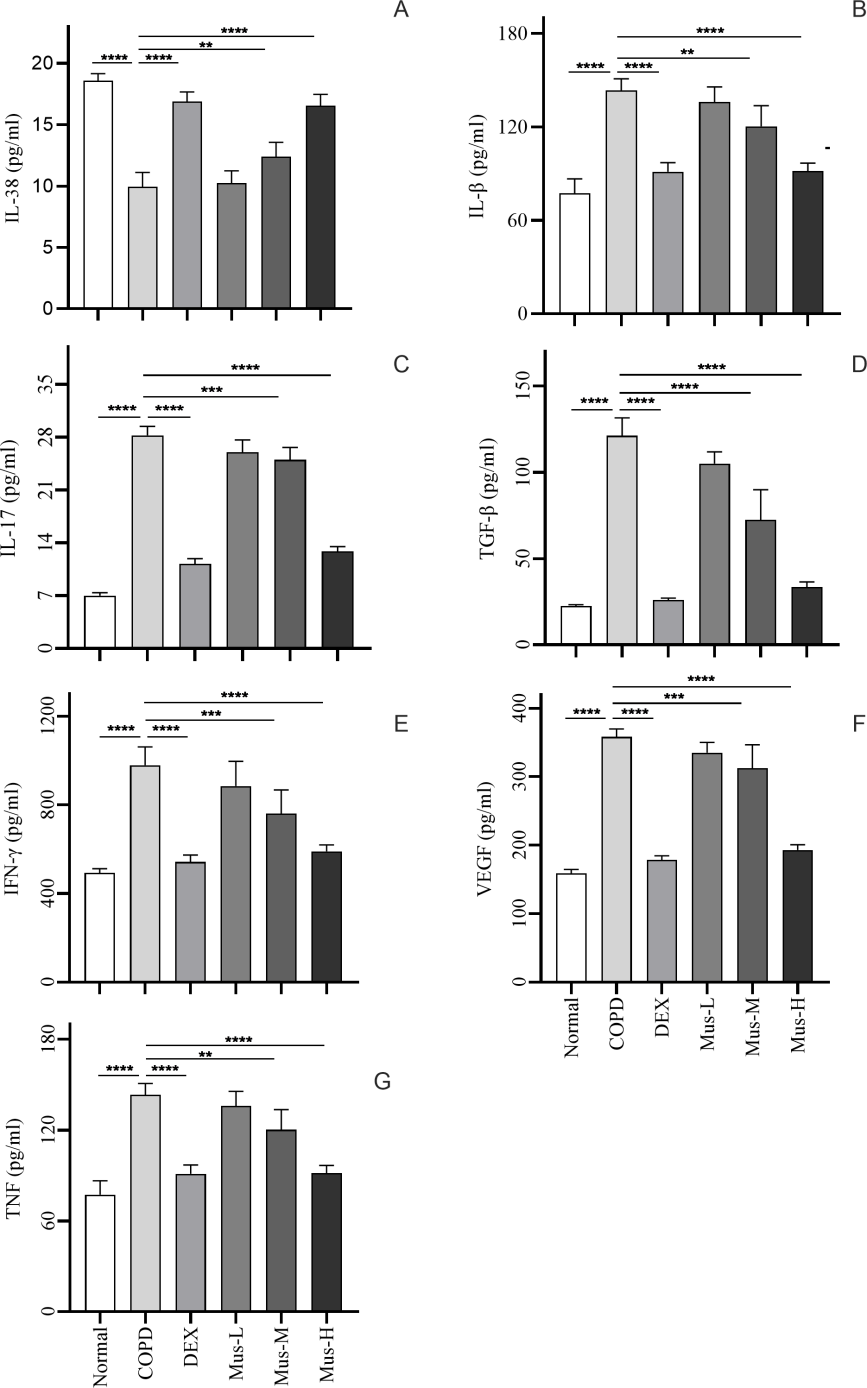
Inflammatory factor expression in serum of mice in each group **(A-G)**. ****P < 0.0001, ***P < 0.001, **P < 0.01.

To investigate the effect of muscone on inflammatory cytokines, we used ELISA to measure serum levels of IL-38 (**Figure 6A**), IL-1β (**Figure 6B**), IL-17 (**Figure 6C**), TGF-β (**Figure 6D**), IFN-γ (**Figure 6E**), VEGF (**Figure 6F**) and TNF (**Figure 6G**).

Serum IL-38 was significantly reduced by approximately 60% in COPD group, compared to that of the normal group (*p* < 0.0001) (**Figure 6A**). DEX almost restored completely the IL-38 from the COPD animals (*p* < 0.0001). Moreover, muscone was also able to restore the suppressed IL-38 in a dose dependent manner. Serum IL-1β, IL-17, TGF-β, IFN-γ, VEGF and TNF (**Figures 6B–G**), were significantly elevated by more than 2 times in the COPD group compared to the normal group (*p* < 0.0001). DEX treatment significantly reduced these mediators in the range of 50-80% compared to mice in the COPD group (*p* < 0.0001). Muscone treatment reduced the serum levels of pro-inflammatory cytokines in COPD animals in a dose-dependent manner.

### Protein levels of CXCR3, IFN-γ, IL-17A and RORγt

**Figure 7:**
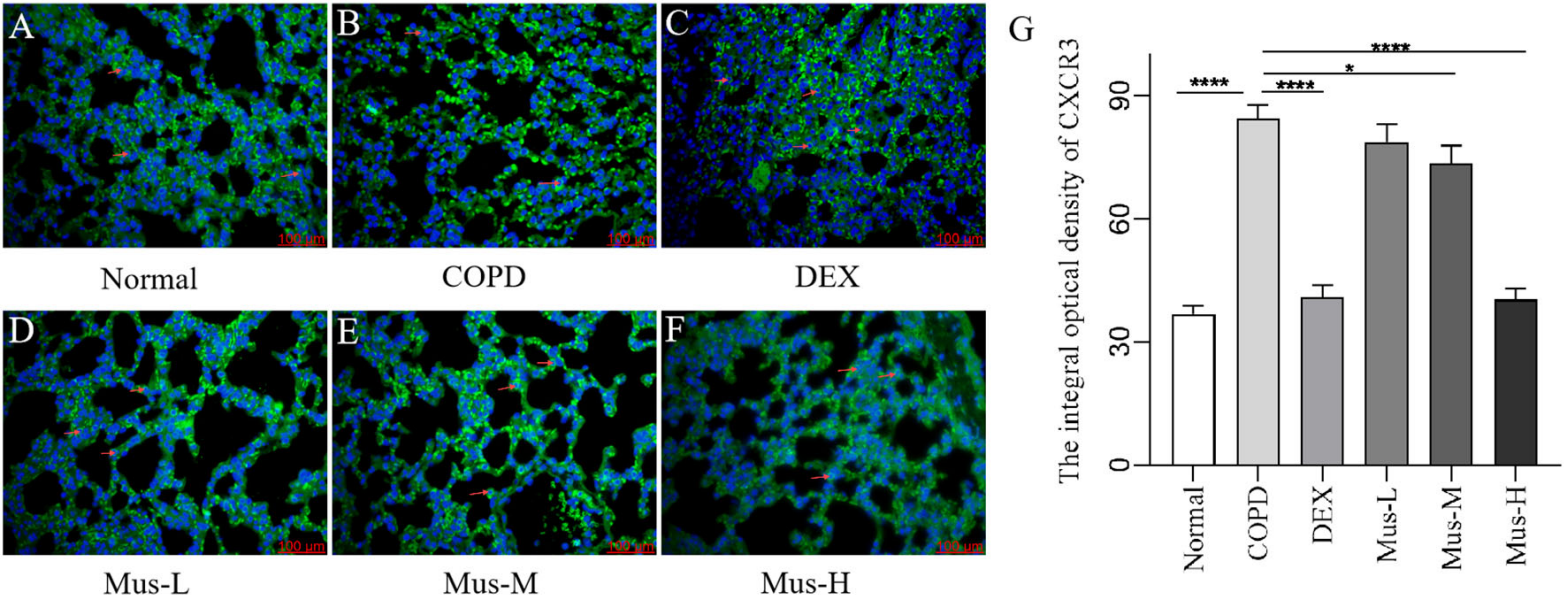
The expression of CXCR3 in lung tissue of mice in each group **(A-G)**. ****P < 0.0001, *P < 0.5.

**Figure 8:**
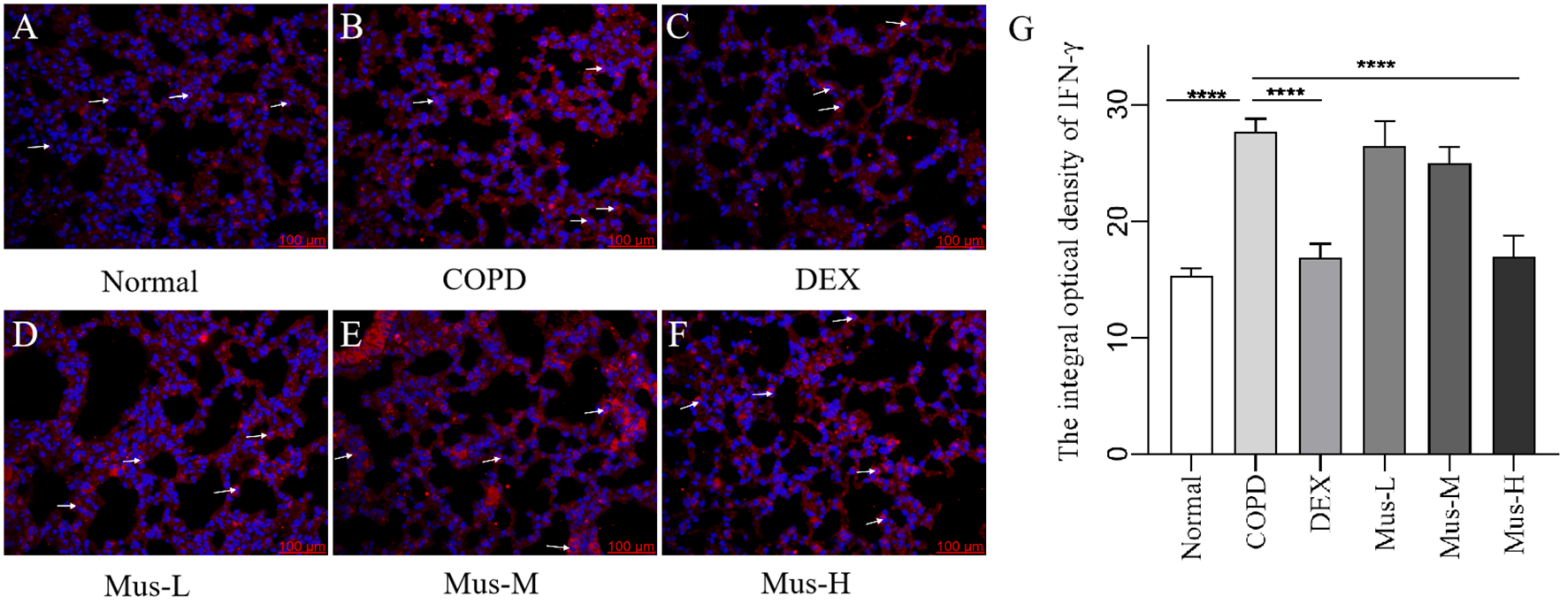
The expression of IFN-γ in lung tissue of mice in each group **(A-G)**. ****P < 0.0001.

**Figure 9:**
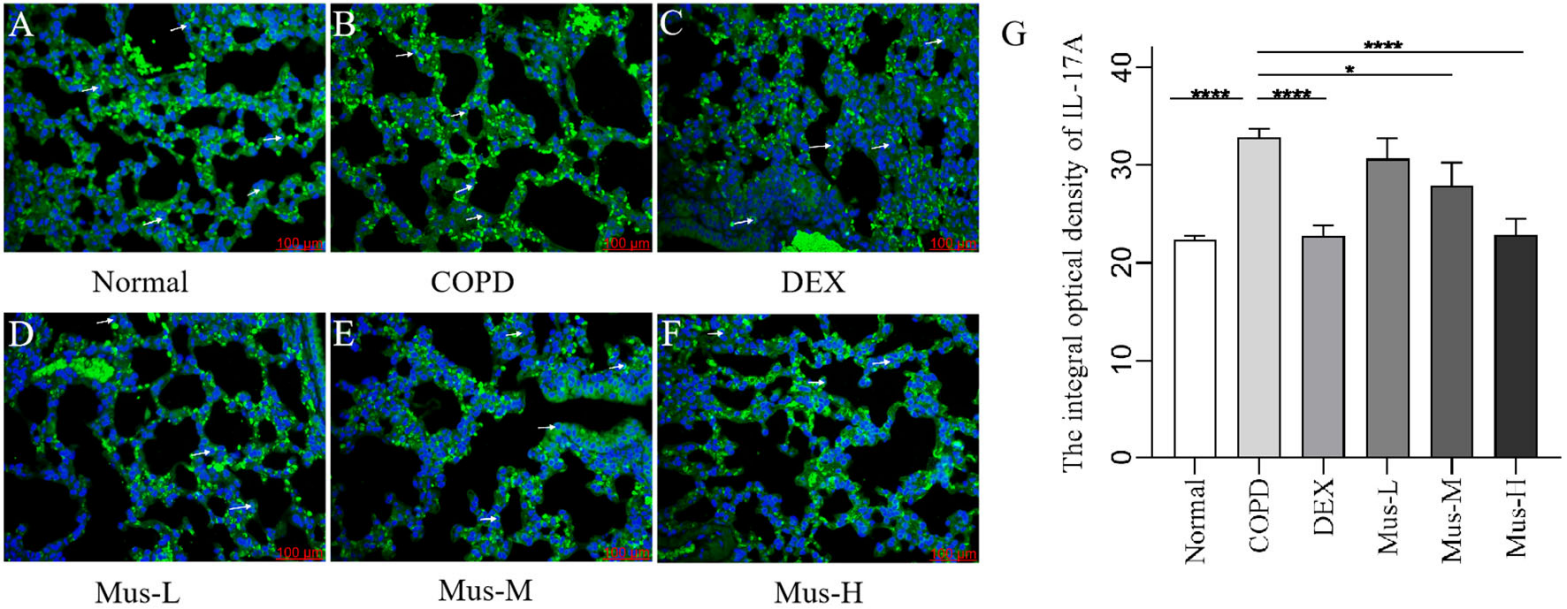
The expression of IL-17A in lung tissue of mice in each group **(A-G)**. ****P < 0.0001, *P < 0.5.

**Figure 10:**
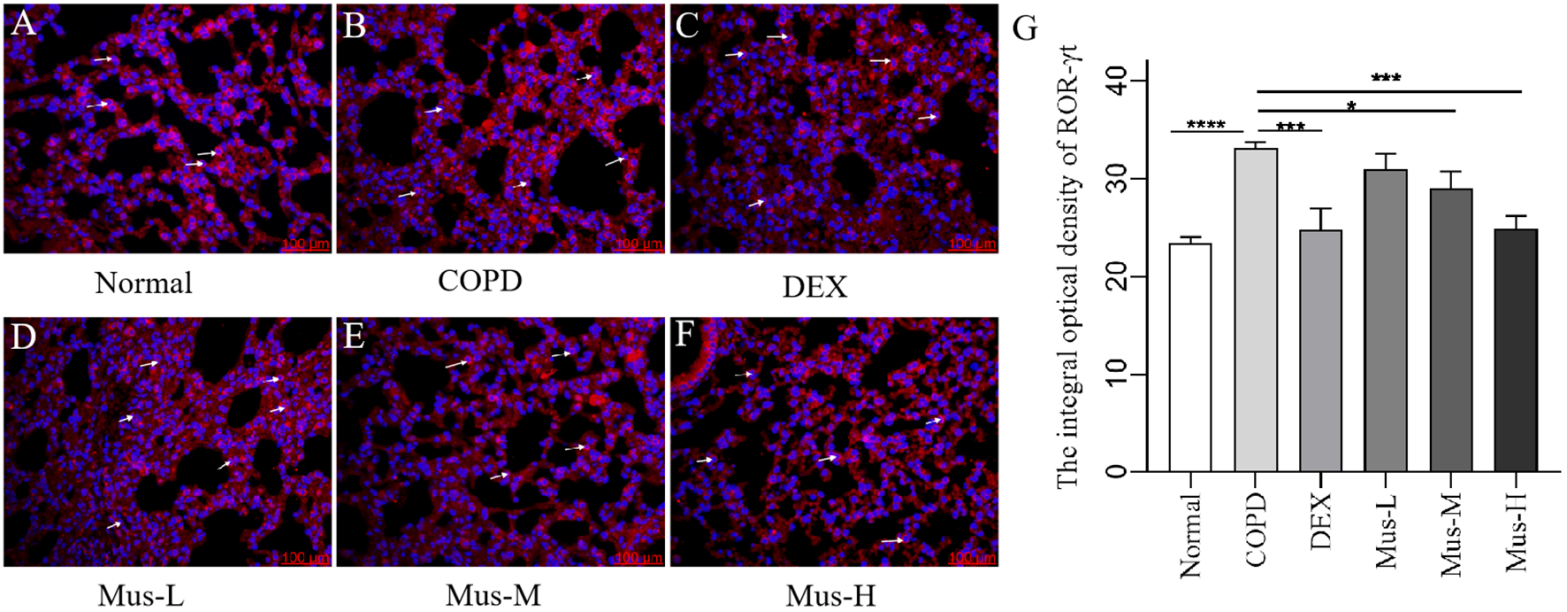
The expression of RORγt in lung tissue of mice in each group **(A-G)**. ****P < 0.0001, ***P < 0.001, *P < 0.5.

To assess the effect of CS+LPS induction on inflammatory factors in mouse lungs, we examined signature inflammatory factor proteins such as CXCR3, IFN-γ secreted by Th1 cells and IL-17A and RORγt secreted by Th2 cells. The CXCR3 results showed that, compared with the normal group (**Figure 7A**), the expression of CXCR3 in the lungs of mice in the COPD model group significantly increased (**Figure 7B**). The expression of CXCR3 was reduced by nearly half after DEX treatment (**Figure 7C**), which showed a significant inhibitory effect. The effect of low-medium- and dose muscone was not as significant as that of the high-dose (**Figures 7D, E**), and the inhibitory effect of high-dose muscone on CXCR3 was basically the same as that of DEX (**Figure 7F**). Optical density analysis showed that the expression of CXCR3 in the lungs of mice in the COPD model group was about 2.5 times higher than that in the normal group (**Figure 7G**), which suggests that severe inflammatory responses accompany the development of COPD. The expression of IFN-γ, IL17A, RORγt in the lungs of animals with COPD and the changes after administration of IFN-γ, IL-17A, RORγt were similar to those of CXCR3 (**Figures 8**–**10**).

## Discussion

This study aimed to investigate the therapeutic effects of Muscone in a mouse model of COPD induced by CS + LPS. A COPD mouse model was established based on previous reports (33). In this study, Muscone was found to alleviate CS + LPS-induced weight loss and pulmonary dysfunction in mice. After two weeks of modeling, the mice exhibited noticeable signs of depression, reduced activity, and significant weight loss. From the third week, their weight began to increase slowly, but after Muscone administration, weight gain accelerated significantly.

Lung function, assessed by measuring parameters such as Ti, Te, MV, TV, PIF, and PEF, is crucial for evaluating COPD severity as it reflects pathological changes in the small airways and is more sensitive than morphological lung changes (34). Following Muscone intervention, lung function parameters significantly improved in COPD mice, indicating that Muscone effectively reversed lung function decline, improved airflow control, and ultimately mitigated emphysema. These findings are consistent with Zhong’s study (35), which reported that muscone improved lung function in patients with pulmonary heart disease.

Histopathological examination of the lungs of COPD mice revealed typical features of the disease, including widened alveolar septa, capillary proliferation and congestion, small areas of hemorrhage, infiltration of lymphocytes, plasma cells, and some neutrophils, partial alveolar collapse, and fusion of a few alveolar cavities. These findings reflect the complex airway and parenchymal changes associated with COPD. Compared to the COPD group, Muscone treatment significantly reduced these histopathological changes, suggesting a protective effect against COPD. This finding aligns with results from other studies (30, 36).

Inflammation plays a crucial role in the pathogenesis of COPD (37). Prolonged exposure to harmful particles triggers immune responses in the respiratory tract, leading to the activation of immune cells and the release of pro-inflammatory cytokines, such as TNF, IL-6, IL-8, and MMPs, which disrupt alveolar structure and contribute to the development of COPD (38).

This study observed reduced circulating and local levels of IL-38 in COPD mice, which were restored by DEX or high-dose Muscone treatment. While IL-38 has been shown to exhibit anti-inflammatory properties in other contexts, its specific role in COPD remains to be fully elucidated.

CS can stimulate alveolar macrophages to release pro-inflammatory cytokines (e.g., IL-1β, TNF) through autocrine and paracrine mechanisms, triggering more severe inflammatory responses by inducing the release of pro-inflammatory and pro-fibrotic cytokines (e.g., IL-17, TGF-β, IFN-γ, VEGF, NLRP3) from recruited neutrophils and T lymphocytes, further exacerbating pulmonary inflammation and fibrosis (39–42). This is consistent with other studies showing that the intensification of the inflammatory response significantly increases the expression levels of IL-1β, IL-17, TGF-β, IFN-γ, VEGF, TNF, and NLRP3 in serum or lung tissue (43). In this study, Muscone treatment significantly inhibited the protein expression of CXCR3, IFN-γ, IL-17A, and RORγt in lung tissues of COPD mice, suggesting a suppression of T cell and macrophage activation.

CXCR3 and IFN-γ are surface markers of Th1 cells, while IL-17A and RORγt are surface markers of Th17 cells. The expression of these markers is directly proportional to the degree of inflammation in COPD (44–47). In COPD patients, T cells in the peripheral airways exhibit an enhanced ability to express CXCR3 and IFN-γ (48), and increased IL-17A secretion from Th17 cells, along with its signature protein RORγt, is directly correlated with the severity of emphysema.

This study has a few limitations. First, only male mice were used to minimize the potential interference of female hormonal fluctuations. However, sex may influence drug response; therefore, we plan to extend the study in the future to include female mice or other populations in a follow-up to fully assess the effects of muscone. Second, the study focused on oral administration of Muscone, without considering potential therapeutic effects through other routes or its potential impact via the nervous system.

## Conclusion

In summary, our study demonstrates that Muscone improves lung function in mice with COPD. Muscone treatment alleviated weight loss, improved lung function, and reduced histopathological changes in the lungs of COPD mice. While the data from our current study suggest a potential correlation between the upregulation of IL-38 and the downregulation of pro-inflammatory cytokines, we do not yet have definitive evidence *in vivo* and/or *in vitro*. This will be confirmed in our future studies.

## Data Availability

The original contributions presented in the study are included in the article/**Supplementary Material**. Further inquiries can be directed to the corresponding authors.
